# Supplementary material for: Mathematical modeling of vancomycin release from Poly-L-Lactic Acid-Coated implants
Source: PLoS One. 2024 Nov 1;19(11):e0311521. doi: 10.1371/journal.pone.0311521 (PMC11530042; doi:10.1371/journal.pone.0311521)
Supplement: S1 File — S1 Fig. Schematic of numerical method for diffusion. S2 Text Description of model derivation. S3.1 Fig. Mesh independent study results where x is the location in the tissue away from the bone plate, and Δx is the mesh size. S3.1 Table Mesh quality of the fluid domain in case of bone plate. S3.2 Fig. Mesh of fluid domain in case of bone plate. S4 Fig. The distribution of scaling factors in the sensitivity analysis. (DOCX) [file pone.0311521.s001.docx]

**Supporting Information**

**S1 Fig:** Schematic of numerical method for diffusion

**S2 Text**

The mass in an element *i* can be written in the term of concentration using **Eq. S2.1**.

**$m_{i}=C_{i}\cdot A\Delta x$ (Equation S2.1)**

At the solid-liquid interface, *i* = 0, the concentration of vancomycin can be calculated from the equation below.

$$\frac{\Delta x}{\Delta t}\left(\left.C_{0}\right|_{t+\Delta t}-\left.C_{0}\right|_{t}\right)=J_{\text{in}}+D\frac{\left(\left.C_{1}\right|_{t}-\left.C_{0}\right|_{t}\right)}{\Delta x}$$

**$\left.C_{0}\right|_{t+\Delta t}=\left.C_{0}\right|_{t}+\frac{\Delta t}{\Delta x^{2}}\left[\Delta x\cdot J_{\text{in}}+D\left(\left.C_{1}\right|_{t}-\left.C_{0}\right|_{t}\right)\right]$ (Equation S2.2)**

The concentration of vancomycin at the element *i* = 1, 2, 3, …, n-1 can be calculated from **Eq. A.3**.

$$\frac{\Delta x}{\Delta t}\left(\left.C_{i}\right|_{t+\Delta t}-\left.C_{i}\right|_{t}\right)=-D\frac{\left(\left.C_{i}\right|_{t}-\left.C_{i-1}\right|_{t}\right)}{\Delta x}+D\frac{\left(\left.C_{i+1}\right|_{t}-\left.C_{i}\right|_{t}\right)}{\Delta x}$$

**$\left.C_{i}\right|_{t+\Delta t}=\left.C_{i}\right|_{t}+\frac{D\Delta t}{\Delta x^{2}}\left[\left.C_{i-1}\right|_{t}-2\left.C_{0}\right|_{t}+\left.C_{i+1}\right|_{t}\right]$ (Equation S2.3)**

At *i* = n; **Eq. A.4** is for the calculation of vancomycin centration.

**$\left.C_{n}\right|_{t+\Delta t}=\left.C_{n}\right|_{t}+\frac{D\Delta t}{\Delta x^{2}}\left[\left.C_{\text{n}-1}\right|_{t}-\left.C_{\text{n}}\right|_{t}\right]$ (Equation S2.4)**

At the beginning of the system, the initial mass of vancomycin can be replaced with the concentration of vancomycin and the volume of each element (*V*).

**$m_{t}=C_{t=0}V\left(\frac{1}{2}\right)^{t/t_{1/2}}$ (Equation S2.6)**

To the estimate the rate of body uptake, the derivatives of each side of the equation with respect to time are taken.

**$\frac{dm_{t}}{dt}=V\cdot C_{t=0}\ln\left(\frac{1}{2}\right)\cdot\left(\frac{1}{2}\right)^{t/t_{1/2}}\cdot\frac{1}{t_{1/2}}$ (Equation S2.7)**

According to the schematic for the numerical calculation in this study, **A.7** is rewritten as:

**$\left.\frac{\Delta m_{t}}{\Delta t}\right|_{\text{Body Uptake}}=A\Delta x\cdot C_{t}\cdot\frac{1}{t_{1/2}}\ln\left(\frac{1}{2}\right)\cdot\left(\frac{1}{2}\right)^{\Delta t/t_{1/2}}$ (Equation S2.8)**

Eventually, the concentration of vancomycin in each element can be estimated by the equations below.

$$\left.C_{0}\right|_{t+\Delta t}=\left.C_{0}\right|_{t}+\frac{\Delta t}{\Delta x^{2}}\left[\Delta x\cdot J_{\text{in}}+D\left(\left.C_{1}\right|_{t}-\left.C_{0}\right|_{t}\right)\right]+\left[\frac{\Delta t\cdot\ln\left(0.5\right)}{t_{1/2}}\cdot\left.C_{0}\right|_{t}\cdot\left(0.5\right)^{\Delta t/t_{1/2}}\right]$$

**(Equation 6)**

$$\left.C_{i}\right|_{t+\Delta t}=\left.C_{i}\right|_{t}+\frac{D\Delta t}{\Delta x^{2}}\left[\left.C_{i-1}\right|_{t}-2\left.C_{0}\right|_{t}+\left.C_{i+1}\right|_{t}\right]+\left[\frac{\Delta t\cdot\ln\left(0.5\right)}{t_{1/2}}\cdot\left.C_{i}\right|_{t}\cdot\left(0.5\right)^{\Delta t/t_{1/2}}\right]$$

**(Equation 7)**

$$\left.C_{n}\right|_{t+\Delta t}=\left.C_{n}\right|_{t}+\frac{D\Delta t}{\Delta x^{2}}\left[\left.C_{\text{n}-1}\right|_{t}-\left.C_{\text{n}}\right|_{t}\right]+\left[\frac{\Delta t\cdot\ln\left(0.5\right)}{t_{1/2}}\cdot\left.C_{n}\right|_{t}\cdot\left(0.5\right)^{\Delta t/t_{1/2}}\right]$$

**(Equation 8)**

**S3 Text**

The mesh independent study is a valuable technique for investigation of the largest element size that can provide reliable results. The one-dimensional diffusion of vancomycin from the bone plate to tissue was used for the analysis. The effect of the mesh size (Δx), varied between 0.05 – 0.2 mm, on the concentration of vancomycin at various location (x = 0.0, 1.5, and 3 mm) at 1 h was investigated (**S3.1**). The results of the study, as shown in **S3.1**, demonstrated that an increase in mesh size from 0.15 mm to 0.2 mm led to fluctuation in the prediction of vancomycin concentration. As a result, a mesh size of 0.15 mm was selected in our subsequent studies, as it consistently yielded stable and reliable results.


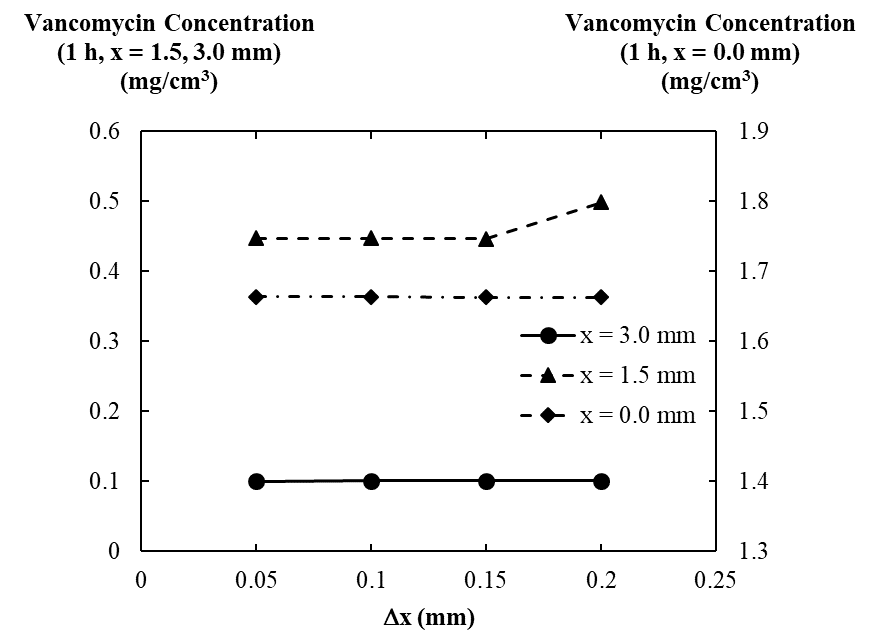


**S3.1 Fig.** Mesh independent study results where x is the location in the tissue away from the bone plate, and Δx is the mesh size.

To ensure reliable simulation results, mesh quality was assessed, considering two key parameters: aspect ratio and face angle. **Table S3.1** shows the mesh quality and the acceptable parameter ranges. An illustration of the 0.15 mm mesh in the fluid domain adjacent to the bone plate is provided in **S3.2**.

**Table S3.1** Mesh quality of the fluid domain in case of bone plate

| Parameters | CFX | Acceptable Range |
| --- | --- | --- |
| Aspect ratio | 1.5 | <100 |
| Face angle (degree) | 90 | 10 - 170 |


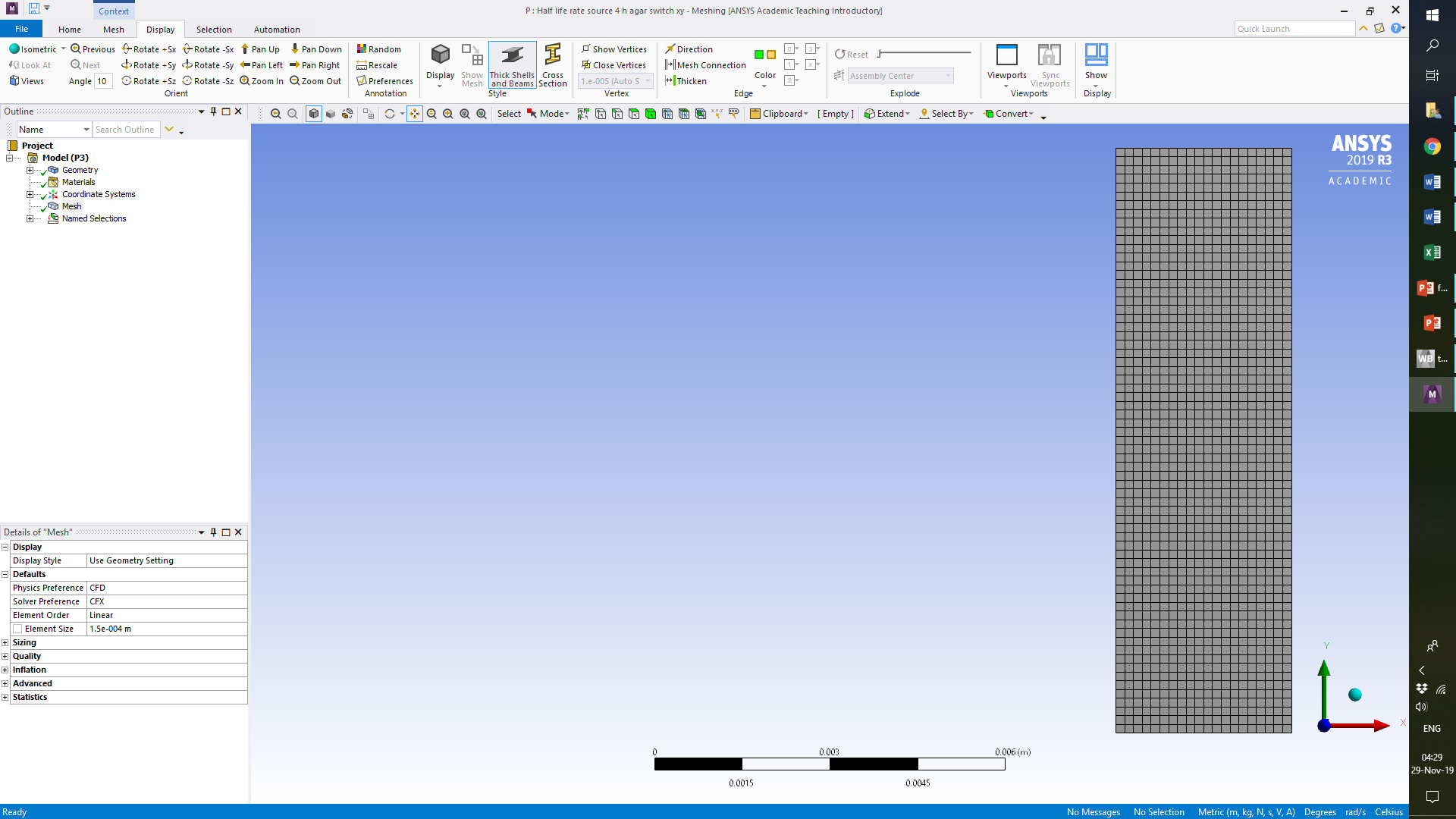


**S3.2 Fig.** Mesh of fluid domain in case of bone plate

**S4**


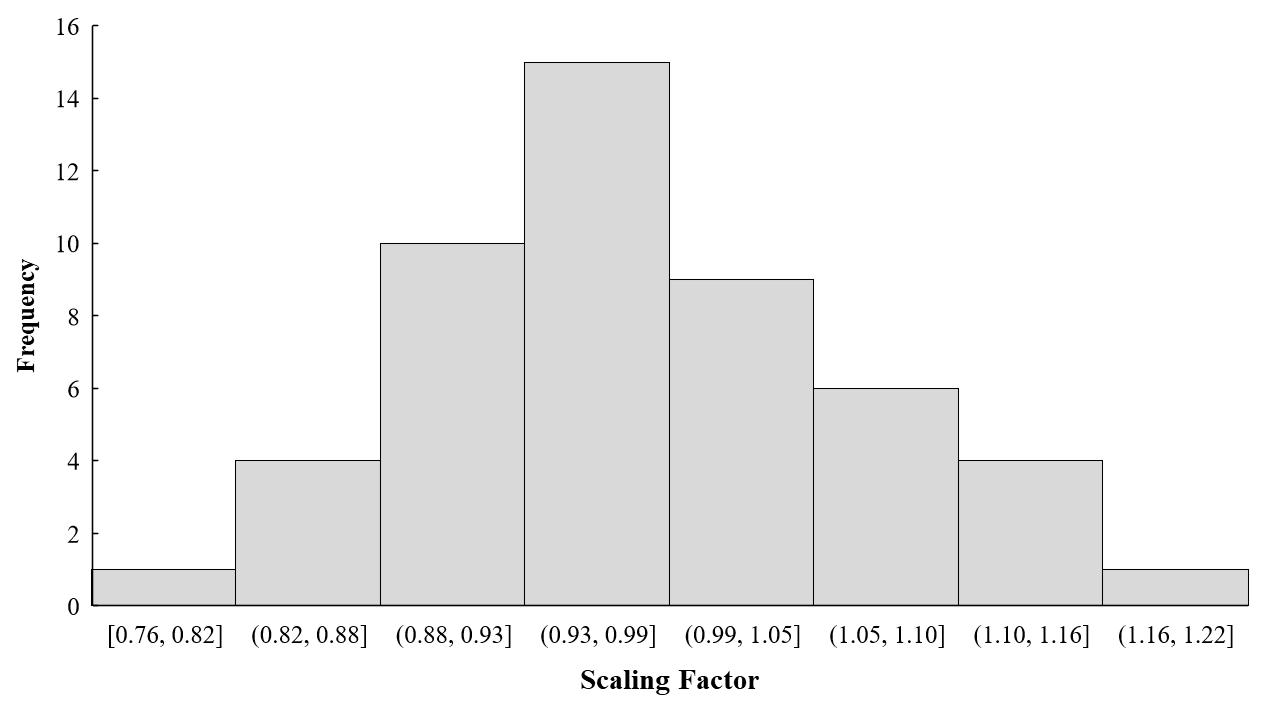


**S4 Fig.** The distribution of scaling factors in the sensitivity analysis
