# Supplementary material for: Mathematical modeling of vancomycin release from Poly-L-Lactic Acid-Coated implants
Source: PLoS One. 2024 Nov 1;19(11):e0311521. doi: 10.1371/journal.pone.0311521 (PMC11530042; doi:10.1371/journal.pone.0311521)
Supplement: Attachment — Submitted filename: Revision about manuscript ID PONE.docx [file pone.0311521.s002.docx]

Revision about manuscript ID PONE-D-24-01184

The manuscript provides a comprehensive computational modeling approach to study the local delivery of vancomycin from a bone plate. The validation of the model using experimental data is supposed to increase the confidence in the simulation results. The sensitivity analysis on the key parameters, such as vancomycin half-life and diffusion coefficients, provides valuable insights into the factors that influence the drug distribution. English is very well written. However, the manuscript could be improved by providing more details on the model development and the assumptions made, as well as a more thorough discussion of the limitations and potential applications of the approach.

The abstract gives the idea that this manuscript performed both mathematical and animal model and compared the results. You should perform a clear text depicting that the comparison is from previous literature already published.

The work was strongly compared to the animal model previous published by Loc-Carrillo et al (2016), reference 14. I have read this literature, and I found some inconsistences in the comparison made when presented in the figure 6 of your manuscript. Additionally, it´s not clear in this figure from which reference you obtained the comparison of the animal study. However, in lines 92-95 and 283-84, you give a support text that bring indicative discussion that Figure 6 was performed using this literature for comparison. The authors should be clear in the description of figure 6 legend, to describe from which reference obtained this comparison. Furthermore, after reading the reference 14, I observed that their release profile *in vivo* presents a heterogenic delivery while in your Figure 6, you presented a continuous homogenous delivery, strongly correlated to your mathematical model. Other important point is that the units obtained in the delivery profile of reference 14 is different (ug/g) from the units you used (ug/mL), how you converted units? Did you consider g and mL as the same thing? Did the research group of reference 14 gave you the converted data? With so, I suggest their citation in acknowledgments. You should explain with more details how obtained the data of your Figure 6 from Fig 1A of Loc-Carrilo (2016). In the topic *Cross-Validation with Published in vivo Data* you discuss about this complexity and data refinement. Thus, I strongly recommend that details about how you processed the data comparison to a very similar mathematical model relating to a literature that presents a different delivery pattern should more well described since this comparison is the most important data of your work.

Despite other minor details on final conclusions, which depicts about “patient safety” in general, the target of this model is to predict the drug effectiveness while when in a delivery PLLA based vancomycin model, thus I recommend depicting more direct outcome such as effective treatment minimizing adverse effects and to avoid ineffective treatment leading to recurrent infection.

After the corrections about the discussion related to figure 6 and abstract rewritten, I recommend to manuscript to be published.
